# Editorial: Biomechanical and cognitive pattern assessment in human-machine collaborative tasks for industrial robotics

**DOI:** 10.3389/fnbot.2026.1858458

**Published:** 2026-05-13

**Authors:** Alessandro Mengarelli, Andrea Tigrini, Ali H. Al-Timemy, Silvia Muceli, Federica Verdini

**Affiliations:** 1 Department of Information Engineering, Università Politecnica delle Marche, Ancona, Italy; 2 College of Excellence, University of Baghdad, Baghdad, Iraq; 3 Department of Electrical Engineering, Chalmers University of Technology, Gothenburg, Sweden; 4 Department of Theoretical and Applied Sciences, eCampus University, Novedrate, Italy

**Keywords:** biomechanics, human-robot interaction, human-robot acceptance, industrial robotic, industry

As collaborative robots have become a central part of modern industry, their value in boosting productivity and sharing workloads with human operators is nowadays well-acknowledged (Xia et al., 2025). However, the growing centrality of human-robot collaboration brings several challenges, because safety, health, and cognitive wellbeing of the working people have to be carefully preserved.

In this Research Topic, the need for a safe and robust evaluation of the human-robot interaction has been tackled by Sunesson et al., who proposed a unified platform for a rigorous evaluation of the interaction between humans and machines when operating in close proximity. Indeed, as robots increasingly share physical spaces with human workers, safety and ergonomics are fundamental requirements for earning trust and enabling true physical human-robot collaboration. The platform design combines physical simulation, haptic feedback, and immersive visual rendering to create an experience that feels real without exposing operators to physical danger. The proposed platform can be used for prototyping, testing, and refining human-robot collaboration strategies without any risk of injury or equipment damage. This contribution promotes research on safety and ergonomics in the field of human-robot interaction and collaboration.

To ensure a safe interaction between humans and robots, a robust pipeline for human motion recognition and prediction can represent a valuable solution. Indeed, as collaborative robots steadily find their place on factory floors and in advanced production lines, a crucial limitation lies in their ability to understand and anticipate human behavior. Pettersson and Falkman addressed this issue by exploiting gaze data to infer human arm movement intentions in virtual reality. Findings of the study are promising in terms of accuracy, prediction and execution time. Therefore, gaze-based intention decoding appears to be a possible way for strengthening and enhancing human-robot collaboration. In fact, if robots can reliably anticipate where a human is about to move, they can better coordinate actions, reduce hesitation, and ultimately make collaborative work both safer and more efficient.

Physical interaction with robots also alters human motor strategies, requiring movements that differ markedly from those used when cooperating with another person. These adapted motor patterns can be less ergonomic and more physically demanding, with potential repercussions for both performance and long-term musculoskeletal health. In fact, the presence of a cobot may reshape how tasks are executed, in ways that are not always benign. Modifications of the biomechanical behavior during human-robot interaction have been analyzed by Ranaldi et al., who considered a sequential collaboration activity. The study showed that trunk motion differed based on the type of interaction. Moreover, leaving most of the task phases to the robot leads to a non-natural way of executing movements for the human. Therefore, during the planning of the robots' actions, a trade-off is recommended between performance improvements and biomechanical comfort of the collaborating human. In this view, an interesting application has been proposed by Jiang and Lu, who proposed a novel cross-modal transfer learning approach for real-time analysis of opponent tactics, that proved to be a suitable solution for an intelligent robot sport competition tactical analysis.

Repeated collaboration with automated systems also influences psychological and cognitive states. Attention, sense of agency, trust, and cognitive load can be reshaped in response to specific interactions, that may cause mental fatigue, reduced vigilance, and increased error rates, undermining performance. This, in turn, might lower the sense of control or comfort, weakening the effectiveness of the collaboration itself. The problem of monitoring human performances, in terms of physical and cognitive assessment has been tackled by Guo et al. Their study focused on the physical interaction between humans and cobots aimed at monitoring health. Specifically, the authors relied on transformer based-multi-modal data fusion for assessing cognitive workload, biomechanical risk factors, and muscular fatigue during human-machine interaction. The proposed approach was tailored to cloud-based robot-assisted systems and achieved remarkable results in grasping the hidden relationship between heterogeneous data modalities, improving safety assessment and accuracy of health monitoring.

Cognitive aspects in human-robot collaboration have been addressed by Someshwar et al., who investigated human-robot collaboration combined with dual-task interference. The latter was designed as an assembly task in collaboration with two robots joined by a cognitive task in a team-work scenario. Overall, outcomes pointed out that gender and age are important factors to take into account in human-robot collaboration, and that collaborative tasks with robots could be designed and assigned to different workers based on their specific and personal skills. Taken together, these factors highlight that the success of collaborative robotics cannot be measured only by throughput or precision but it hinges equally on understanding how such systems reshape human behavior from the physical, cognitive, and communicative viewpoint. For instance, interaction with robotic agents also influences the communication behavior in humans, potentially leading to different schemes and habits.

Marmor et al. investigated remote communication between telepresent people, and physically present people when mediated by a telepresence robot. The study focused on the spatial behavior of the telepresent person during their interaction with the physically present ones, with the aim to understand the level of the interaction productivity, and the pleasantness perceived by the participants. The study highlighted significant differences in the way physically present people interact with telepresent individuals and vice versa, indicating that communication mediated by telepresence robots shapes the physical interaction between the actors involved. This, in turn, drives the quality and characteristics of the communication itself.

Finally, this Research Topic highlighted also the importance of user requirements for easing interaction with robots and promoting this kind of technology in multiple domains. For instance, Louca et al. conducted interviews with 13 expert operators across four domains, i.e., nuclear maintenance, surgical robotics, underwater exploration, and ordnance disposal, focusing on the aspects that drive the trustworthiness in this kind of systems. Outcomes showed that across all fields except surgery, operators prioritize a comprehensive engineering understanding of the system itself rather than advanced automation or sophisticated assistance features. Other requirements identified by the study were the improvement of situational awareness, and the need for easing the operator training, by including different levels of risk and realism. For instance, the user should be allowed to practice for a large amount of time, in particular when operating systems that present rare faults or undesired movements.
